# A Purification Method of ^18^F-FP-(+)-DTBZ *via* Solid-Phase Extraction With Combined Cartridges

**DOI:** 10.3389/fmed.2021.693632

**Published:** 2021-07-09

**Authors:** Yuyin Dai, Ri Sa, Feng Guan, Qi Wang, Yinghua Li, Hongguang Zhao

**Affiliations:** Department of Nuclear Medicine, The First Hospital of Jilin University, Changchun, China

**Keywords:** ^18^F-FP-(+)-DTBZ, 18F-FDG, PET/CT, solid phase extraction, Parkinson disease

## Abstract

**Background:** To optimize [^18^F] 9-fluoropropyl-(+)-dihydrotetrabenazine (^18^F-FP-(+)-DTBZ) purification via solid-phase extraction (SPE) with combined cartridges to facilitate its widespread clinical application.

**Methods:** A modified SPE purification method, employing Sep-Pak PS-2 and Sep-Pak C18 cartridges, was used for the preparation of ^18^F-FP-(+)-DTBZ. This method was compared to the purification method of high-pressure liquid chromatography (HPLC) and SPE with one cartridge, following quality control test and positron emission tomography (PET) imaging in healthy volunteers and patients with parkinsn's disease (PD).

**Results:** A SPE purification method integrating Sep-Pak PS-2 and Sep-Pak C18 cartridges was implemented successfully. The retention time of ^18^F-FP-(+)-DTBZ purified by HPLC, SPE with Sep-Pak PS-2, SPE with Sep-Pak C18, and SPE with combined use of Sep-Pak PS-2 and Sep-Pak C18 cartridges was 8.7, 8.8, 8.7, and 8.9 min, respectively. Fewest impurity peak was detected in ^18^F-FP-(+)-DTBZ purified by the SPE with combined use of Sep-Pak PS-2 and Sep-Pak C18 cartridges. This modified SPE purification method provided a satisfactory radiochemical yield of 29 ± 1.8% with radiochemical purity >99% and shortened synthesis time to 27 min. The brain uptake of ^18^F-FP-(+)-DTBZ purified by the modified SPE was comparable to that purified by HPLC in both healthy volunteers and PD patients.

**Conclusions:** A SPE method integrating Sep-Pak PS-2 and Sep-Pak C18 cartridges for purification of ^18^F-FP-(+)-DTBZ may be highly suited to automatic synthesis for routine clinical applications, as it provides excellent radiochemical purity, high yield as well as operational simplicity.

## Introduction

Vesicular monoamine transporter type 2 (VMAT2) plays critical role in the mechanism of packaging and transporting neurotransmitters from the cytosol into synaptic vesicle, verifying its status as an objective marker of nigrostriatal terminal integrity (1, 2). VMAT2 located on vesicle membranes in neurons and its activity largely affects the scale of dopamine release (3). The activity of the presynaptic monoaminergic binding site is less likely influenced by the medication or compensatory mechanisms, as it is active only when VMAT2-containing neurons are active (4). Therefore, it is a reliable and objective biomarker for exploring neurological and psychiatric disorders such as Parkinson disease (PD) (5), Alzheimer's disease and dementia with Lewy bodies (6), as well as endocrine system disease such as diabetes (7–9).

The imaging of VMAT2 has potential advantages such as better image quality and quantification, shorter time between tracer administration and scanning, shorter scan duration, and no requirement for prior blockade of the thyroid to prevent radioactive iodine uptake (10–15). To date, different kinds of agents for VMAT2 have been investigated to map VMAT2 distribution, and some of them have been tested in humans and achieved favorable results (16–18). For example, as early as in 1996, Frey et al. (19) found that PD patients had reduction in specific [^11^C]dihydrotetrabenazine (DTBZ) distribution volume in the putamen (−61%) and in the caudate nucleus (−43%). Similarly, regional specific uptake ratio of [^18^F] 9-fluoropropyl-(+)- DTBZ (^18^F-FP-(+)-DTBZ) of caudate, putamen, substantia nigra, and globus pallidus in the moderate and advanced PD patients were significantly lower than those in the healthy human subjects (16). More encouragingly, Li et al. (8) provided evidence that dysfunction of streptozotocin-diabetic retinas was detected by ^18^F-FP-(+)-DTBZ imaging at least 4 weeks earlier than other examinations such as electroretinogram, color fundus photography, and angiography. Generally, widespread use of this agent could facilitate the disease diagnosis and monitoring treatment response.

However, the published methods of purification of this agent cost a long preparation time, which limits its routine application for VMAT2 tracing. High-pressure liquid chromatography (HPLC) is a conventional method used for purification of ^11^C-labeled or ^18^F-labeled DTBZ to eliminate the pseudo-carrier. The procedure of this purification method was relatively complex, and the preparation of clinically useful radiotracer using HPLC lasted over 1 h. Subsequently, solid phase extraction (SPE) were adopted and the shortest synthesis time for ^18^F-FP-(+)-DTBZ using SPE was 40 min in the published studies (20, 21). Therefore, purification of ^18^F-FP-(+)-DTBZ is in need to improve in order to increase the application of this tracer. In the present study, we employed a SPE method with combined use of Sep-Pak PS-2 and Sep-Pak C18 cartridges to purify ^18^F-FP-(+)-DTBZ to maximize the final yield and simplify the purification process. With this method, ^18^F-FP-(+)-DTBZ can be expanded into the clinical setting to test the usefulness of ^18^F-FP-(+)-DTBZ in the diagnosis and monitoring of diseases.

## Materials and Methods

### Chemicals and Reagents

All reagents were commercially acquired and utilized without further purification unless otherwise stated. 4,7,13,16,21,24-Hexaoxa-1,10-diazabicyclo [8.8.8] hexacosane (K222, 98.0 %) was purchased from ABX (Germany). Anhydrous acetonitrile and potassium carbonate (K_2_CO_3_) were purchased from Sigma (America). All SPE cartridges were purchased from Waters Corporation (USA). To activate Sep-Pak QMA, Sep-Pak C18, and Sep-Pak PS-2, we flushed them with 5 mL absolute ethanol followed by 10 mL water.

### Radiosynthesis of ^18^F-FP-(+)-DTBZ

**Figure 1:**
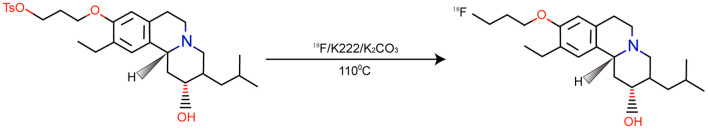
Synthesis route of ^18^F-FP-(+)-DTBZ.

**Figure 2:**
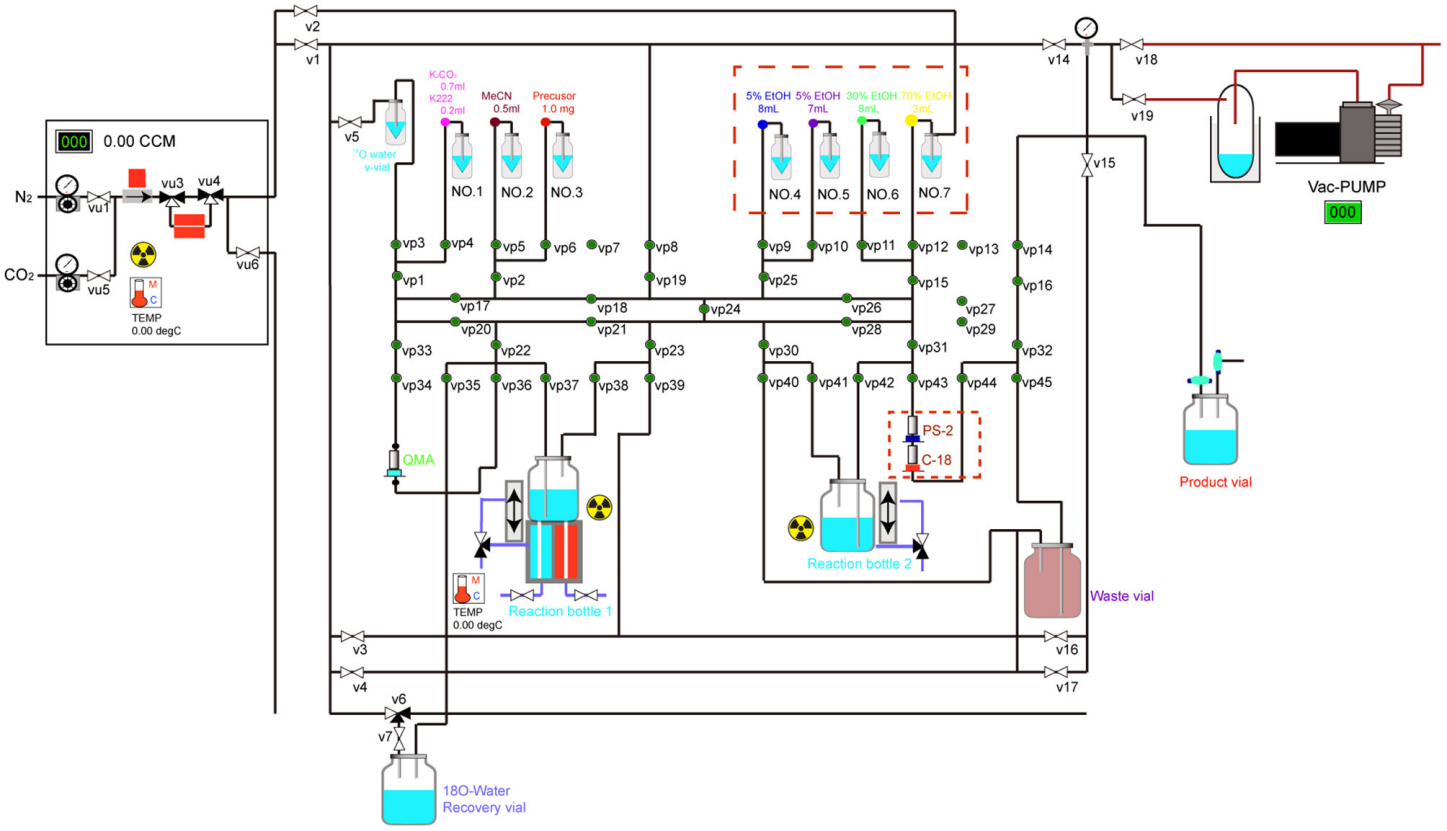
Diagram of the automated system of radiosynthesis of ^18^F-FP-(+)-DTBZ.

Precursor of ^18^F-FP-(+)-DTBZ was synthesized in the College of Chemistry, Jilin University (Figure 1). ^18^F was produced in a cyclotron by ^18^O (p, n)^18^F nuclear reaction. ^18^F was passed into the CFN-MPS200 multi-functional synthesis module through the target transmission system and captured by the Sep-Pak QMA anion exchange column. The residual liquid was put into the oxygen water recovery unit. QMA was eluted with a 0.9mL K2.2.2 / K_2_CO_3_ mixture. Then, ^18^F was rinsed into the reaction bottle. The eluent was dried through successive evaporation with nitrogen (N_2_) flow (30 ml/min) at 100°C for 3 min to remove water and acetonitrile. Next, the residue was redried under the same N_2_ flow at 110°C for 2 min. A total of 1.0 mg TsOP-(+)-DTBZ precursor solution in anhydrous was introduced into the reaction tube. Then, radiofluorination was performed in the reaction bottle at 120°C for 12 min and cooled to 40°C. We transferred the mixture from the reaction bottle to the transfer bottle. The reaction bottle was cleaned using 5 mL 5% ethanol solution, which was then poured into the transfer bottle. The crude product solution purification by HPLC or SPE and the final solution in the collection bottle was passed through a Millex GV for sterilization and diluted to 10 mL with saline in a sterile bottle. The synthesis route and diagram of the automated system of ^18^F-FP-(+)-DTBZ are outlined in Figure 2.

### HPLC Purification of ^18^F-FP-(+)-DTBZ

After radiofluorination, we transferred the crude product to preparative HPLC (mobile phase of acetonitrile: 50 mM ammonium acetate = 57:43; flow 4 ml/min, velocity; 280 nm, ultraviolet absorption wavelength; ultraviolet absorption peak in 12 + 2 min, plus or minus 2 min 14 radiation peak, collect radiation peak), and then absorbed on C18 column after dilution with saline solution. Exactly 1.5 ml of ethanol was to elute the C18 column. Ethanol was diluted to 10% concentration using 0.9% sodium chloride. The final products of ^18^F-FP-(+)-DTBZ were obtained following aseptic filtration.

### SPE Purification of ^18^F-FP-(+)-DTBZ

**Figure 3:**
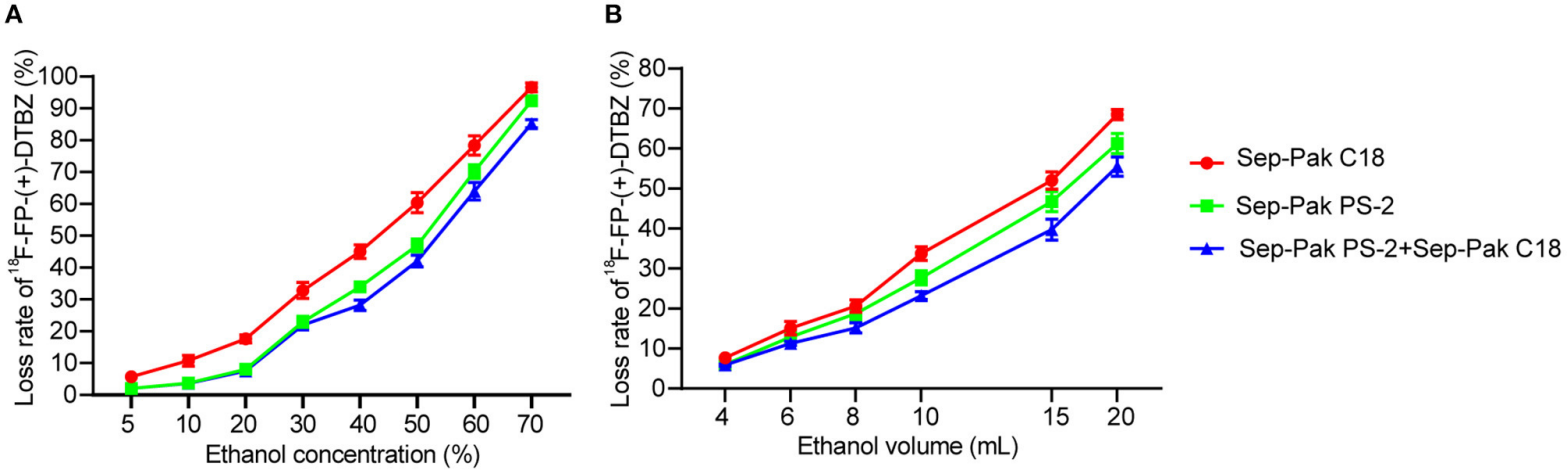
Loss rate of ^18^F-FP-(+)-DTBZ using Sep-Pak PS-2 cartridge and/or Sep-Pak C18 cartridges under different concentration of 10 mL ethanol elusion **(A)** and different volume of 30% ethanol elusion **(B)**.

The crude product solution was passed through the small column of Sep-Pak PS-2 and/or Sep-Pak C18 to eliminate unreacted [^18^F] fluoride and water-soluble impurities, which finally was channeled to the waste liquid bottle. To determine the optimum concentration of elusion ethanol, we used 10 mL ethanol with different concentrations from 5 to 70% to wash out the impurities, and found that the lost rate was in acceptable range when the ethanol concentration was below 30% (Figure 3A). Similarly, to determine the optimum volume of elusion ethanol, we used 30% ethanol with different volumes from 4 to 20 mL of ethanol to wash out impurities, and found the loss rate was in acceptable range when using 10 mL ethanol or below 10 mL. The best final condition for elution ethanol was as follows: for the first time, 7 mL 5% ethanol solution was used to wash out impurities to the waste vial; for the second time, 8 mL 5% ethanol solution was used to wash out excess impurities to the waste vial; for the third time, 8 mL 30% ethanol solution was used for further removal of impurities to the waste vial. Then, the final product was eluted with 3 mL 70% ethanol solution to the product vial as 70% ethanol was enough to elute the all product (Figure 3B).

### Quality Control of ^18^F-FP-(+)-DTBZ

#### Visual Inspection

The ^18^F-FP-(+)-DTBZ product solution was visually examined in the labeling hot cell through the leading glass window under bright light.

#### pH

We employed the pH indicator paper (pH 2–9) referring to the attached indicator chart to estimate the pH value of the purified ^18^F-FP-(+)-DTBZ product.

#### Radiochemical Purity

Before purity analysis, a reference substance solution (non-radioactive FP-(+)-DTBZ; 20 μl, 2.7 mmol/L) was injected into the analytical HPLC system. The chemical purity of ^18^F-FP-(+)-DTBZ after purification was determined by HPLC with UV detection at 280 nm and radiation purity of the product was monitored by HPLC with radiation detector. Simultaneously, thin layer chromatography (TLC) was also applied to the determine the radiation purity.

#### Bacterial Endotoxin Test

Limulus Amebocyte Lysate (LAL) assay was applied to test for bacterial endotoxins.

#### Imaging Quality

Two healthy volunteers and two PD patients underwent PET/CT scan to test the imaging quality of ^18^F-FP-(+)-DTBZ from HPLC and SPE with combined use of Sep-Pak PS-2 and Sep-Pak C18 cartridges. This study was approved by the Ethics Committee of the First Hospital of Jilin University (20K070-001) and conducted in accordance with the 1964 Declaration of Helsinki and its later amendments or comparable ethical standards. Patients were recruited for enrollment in this study from December 2020 through January 2021 at our institute, with agreement from the oncologists and on determination of the patients' eligibility.

## Results

The loss rate of SPE with combined use of Sep-Pak PS-2 and Sep-Pak C18 cartridges was lower than that of SPE with one cartridge at the same concentration of ethanol and same volume of ethanol elusion (Figure 3).

**Figure 4:**
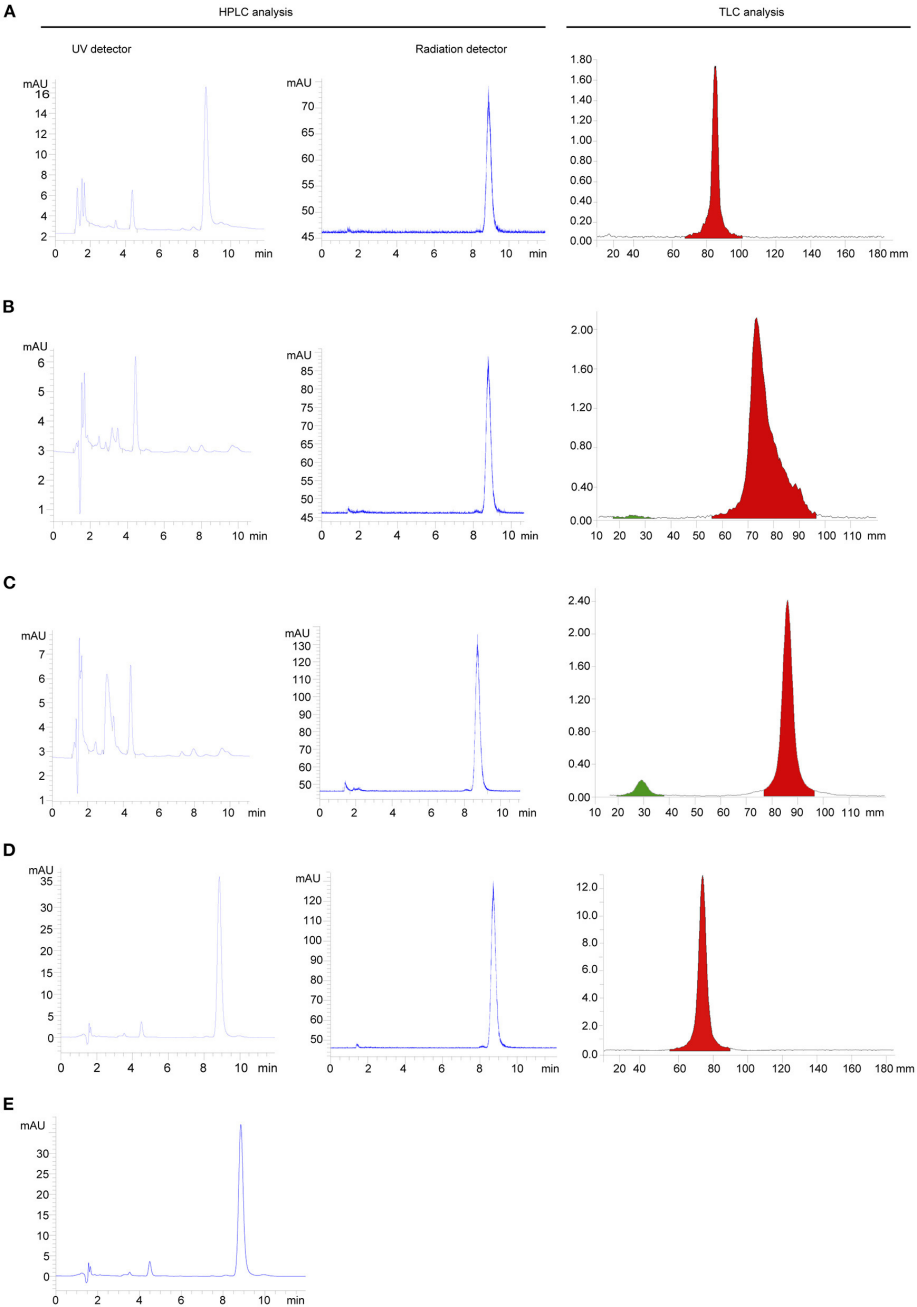
HPLC and TLC analysis of radiochemical purity of ^18^F-FP-(+)-DTBZ purified by HPLC **(A)**, SPE with Sep-Pak PS-2 **(B)**, SPE with Sep-Pak C18 **(C)** SPE with combined use of Sep-Pak PS-2 and Sep-Pak C18 **(D)**, and radiochemical purity of FP-(+)-DTBZ **(E)**.

The identity of ^18^F- FP-(+)-DTBZ was confirmed by comparing the retention time of the radioactive product with that of FP-(+)-DTB. The retention time of ^18^F- FP-(+)-DTBZ purified by HPLC, SPE with Sep-Pak PS-2, SPE with Sep-Pak C18, and SPE with combined use of Sep-Pak PS-2 and Sep-Pak C18 cartridges was 8.7, 8.8, 8.7, and 8.9 min, respectively. Either ^18^F-FP-(+)-DTBZ purified by HPLC or SPE matched well with that of FP-(+)-DTBZ within an admissible error. Fewest impurity peak was detected in ^18^F-FP-(+)-DTBZ purified by SPE with combined use of Sep-Pak PS-2 and Sep-Pak C18 cartridges in all purification methods (Figure 4).

**Figure 5:**
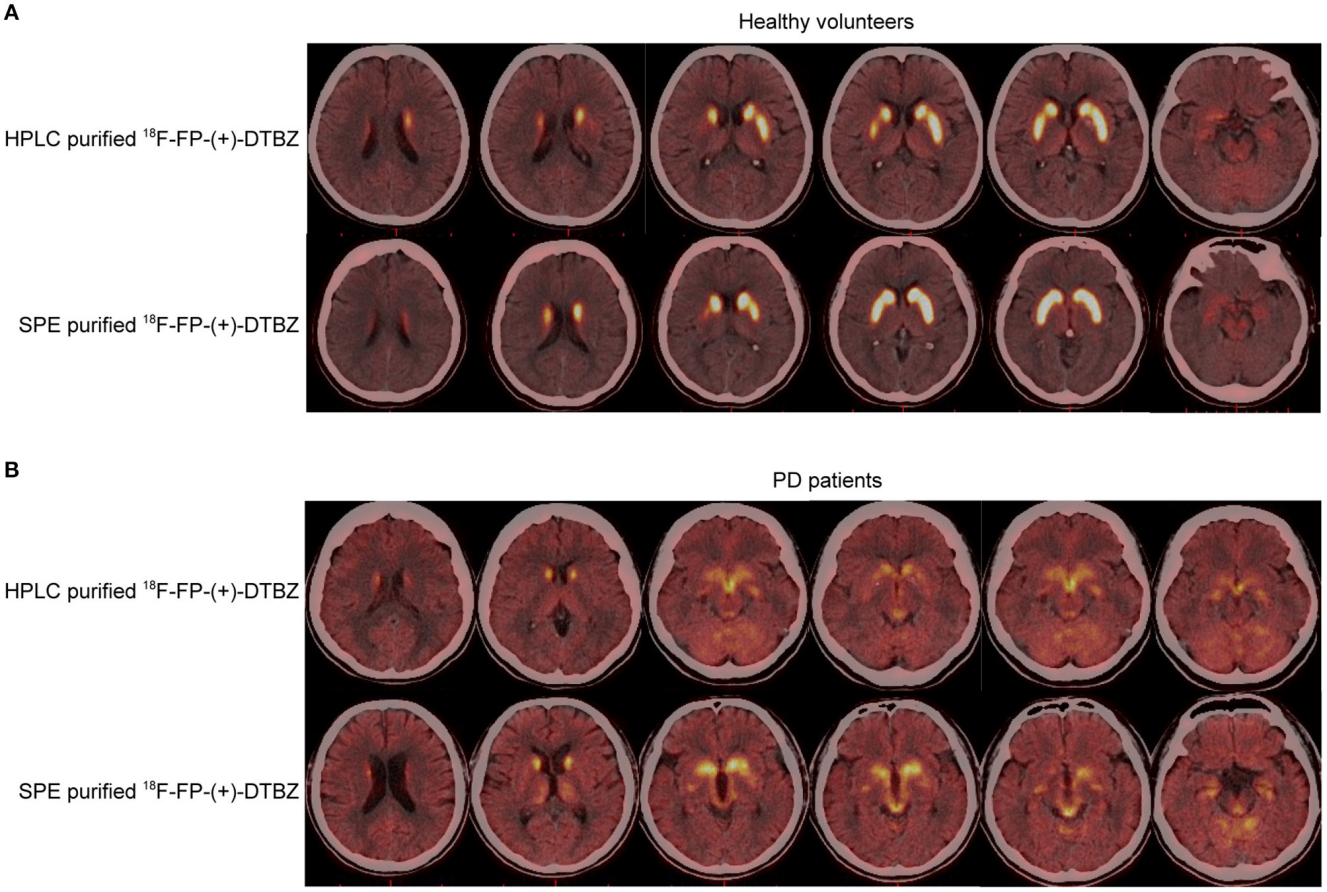
PET/CT performance of HPLC- and SPE- purified ^18^F-FP-(+)-DTBZ in healthy volunteers **(A)**, and PD patients **(B)**.

Comparison of HPLC and SPE purification method of ^18^F-FP-(+)-DTBZ is presented in Table 1. The radiochemical purity exceeded 99%, and the product was radiochemically stable for at least 3 h for the tracers purified by HPLC and SPE with combined use of Sep-Pak PS-2 and Sep-Pak C18 cartridges. The synthesis time of ^18^F-FP-(+)-DTBZ with SPE purification was significantly shorter than that with HPLC purification. Brain uptake of ^18^F-FP-(+)-DTBZ purified by SPE with combined use of Sep-Pak PS-2 and Sep-Pak C18 cartridges was comparable to that purified by HPLC in both healthy volunteers and PD patients (Figure 5). In healthy volunteers, a symmetric distribution pattern of ^18^F-FP-(+)-DTBZ was shown in caudate, putamen, globus pallidus, substantia nigra. In PD patients, ^18^F-FP-(+)-DTBZ avidity in nigrostriatal regions reduced obviously (Figure 5).

**Table 1:** Comparison between HPLC and SPE purification of ^18^F-FP-(+)-DTBZ.

	**HPLC**	**SPE**		
**Column**	**YMC-Pack ODS-AM**	**Sep-Pak C18**	**Sep-Pak PS-2**	**Sep-Pak C18, Sep-Pak PS-2**
Precusor (mg)	1	1	1	1
Procedure synthesis time (min)	65	27	26	27
Synthesis times	3	3	3	5
Yield (GBq)	4.6 ± 5.7%	4.8 ± 8.3%	5.0 ± 10.2%	5.1 ± 7.9%
Yield rate	11 ± 2.3%	25 ± 2.3	27 ± 2.1	29 ± 1.8 %
Activity concentration at the end of synthesis (MBq/mL)	2.3 × 10^3^	1.5 × 10^3^	1.7 × 10^3^	1.7 × 10^3^
Appearance (visual inspection)	Clear, transparent, free of impurities	Clear, transparent, free of impurities	Clear, transparent, free of impurities	Clear, transparent, free of impurities
PH	6–7	6–7	6–7	6–7
Radiochemical purity (radio-HPLC)	99.2%	98.5%	98.0%	99.0%
Radiochemical purity (radio-HPLC) after 3 half time	96.3%	95.6%	95.1%	96.0%
Bacterial endotoxin test (EU/ml)	<15	<15	<15	<15
K2.2.2 comparing to standard reference	less than standard K2.2.2 spots	less than standard K2.2.2 spots	lessthan standard K2.2.2 spots	less than standard K2.2.2 spots
Retention time (min)	8.7	8.8	8.7	8.9

*HPLC, high pressure liquid chromatography; SPE, solid-phase extraction*.

## Discussion

In this study, we optimized SPE purification of ^18^F-FP-(+)-DTBZ via combining Sep-Pak PS-2 and Sep-Pak C18 cartridges. The radiochemical purity from this modified SPE purification method was much more ideal for clinical use than those purified by HPLC or SPE with one cartridge. By testing the imaging quality of ^18^F-FP-(+)-DTBZ in healthy volunteers and PD patients, we further elucidated that the imaging quality of ^18^F-FP-(+)-DTBZ from the modified SPE was comparable to that from HPLC. Additionally, the preparation time of ^18^F-FP-(+)-DTBZ synthesis was shortened to 27 minutes via use of the modified SPE purification.

In the early 1990s, ^11^C labeled tetrabenazine (TBZ), dihydrotetrabenazine (DTBZ), and methoxytetrabenazine (MTBZ) were continuously investigated as tracers for imaging of VMAT2 (22, 23). At that time, silica gel semi-preparative HPLC was mainly adopted in the purification of different kinds of tracers. ^11^C-TBZOMe was purified using a silica gel semi-preparative HPLC column, by passing the HPLC solvent (CH2CI2: hexane: (isopropanol: diethylamine 24:l) 37:62:1; 6 mL/min) through the extraction column and onto the HPLC column (24). Similarly, in the preparation of ^11^C-TBZ and ^11^C-DTBZ, the radiotracer was also purified using semipreparative silica gel chromatography and prepared for injection by evaporation of the HPLC solvent (23, 25). Considering the half time of radionuclide, ^18^F-labeled DTBZ analogs (T1/2 = 110 min) will be more practical to increase the application in clinics equipped with PET scanners but not with sufficient resources to make ^11^C (T1/2 = 20 min) (26, 27). Radiosynthesis of ^18^F-FP-(+)-DTBZ was firstly reported in 2006 (26). It is prepared with ^18^F fluoride as a substitute for the O-tosylate leaving group of precursors, catalyzed by K222/K_2_CO_3_ following the purification of the reaction mixture via semipreparative HPLC column. The preparation of ^18^F labeled compound took about 50–55 min at the end of synthesis.

Since HPLC is a long and laborious process, accompanied by considerable loss of radioactive product, multiple sequential solid-phase cartridges were, therefore, adopted to optimize the preparation of different radiotracers in the early 2010s (20, 21, 28). It is worth noting that the preparation time distinguishes HPLC- from SPE-purified ^18^F-FP-(+)-DTBZ. For instance, in 2010, Zhu et al. employed different precursors (-OTs or -Br as the leaving group at the 9-propoxy position), reagents [K222/K(2)CO(3) vs. tributylammonium bicarbonate] and solvents (acetonitrile vs. DMSO), reaction temperature, reaction time, and purification method with SPE (Oasis HLB) to improve ^18^F-FP-(+)-DTBZ radiosynthesis. This method generated a satisfactory radiochemical yield of 21–41% (*n* = 10) with radiochemical purity > 95% and shortened the synthesis time to 40 min (20). In 2014, a new ^11^C-labeled radiotracer, 10-(11) C-dihydrotetrabenazine (10-^11^C-DTBZ) was successfully synthesized and purified by SPE using an alumina Sep-Pak cartridge, the final 10-^11^C-DTBZ product was obtained. The overall synthesis time was only 20 min from bombardment to release of the product for quality control (29). In 2019, SPE with Sep-Pak C18 cartridge was implemented into the purification of the ^18^F-FP-(+)-DTBZ (30). In this study, they have tested purification effect of SPE equipped with different cartridges including Sep-Pak Oasis, tC2, C8, tC18, CN, Chromafix C18 Hydrox, Chromafix C18, and Chromfix C4, and finally focused on the Sep-Pak tC18 with suitable ethanol-water elution as this system showed the smallest loss in activity. The desired product was obtained in 35 min. In 2020, Zhao et al. (31) successfully obtained a radiolabeling method of ^18^F-FP-(+)-DTBZ under the optimized conditions (P/K2CO3 = 1:8, heating at 120°C for 3 min in dimethyl sulfoxide). The automated synthesis gave a high activity yield of 30–55% in about 40 min with a >99.0% radiochemical purity. Taken together, using SPE instead of HPLC for purification is the trend for different kinds of radiotracers.

Herein, we modified the SPE purification equipped with two different cartridges to obtain four goals: eliminate the residual chemical impurities, achieve excellent radiochemical purity, maximize the final product, and simplified purification method. In the previous studies, SPE with one cartridge was used for the purification of ^18^F-FP-(+)-DTBZ. However, SPE with two cartridges have not been reported. In our study, we have compared the loss rate and radiochemical purity of ^18^F-FP-(+)-DTBZ from SPE with two cartridges to those from SPE with one cartridge. Of note, SPE with two cartridges showed lower loss rate than SPE with one cartridge under the same concentration or same volume of ethanal elution. Whereas, under the same elution condition, although radiation purity of product from SPE with one cartridge was comparable to that from SPE with combined cartridges in the analysis of analytic HPLC with radiation detector, both HPLC analysis with UV detector and TLC analysis showed fewer impurity peaks in ^18^F-FP-(+)-DTBZ from SPE with two cartridges comparing to that from SPE with one cartridge. Therefore, considering the loss rate, radiochemical purity and final yield, ^18^F-FP-(+)-DTBZ from SPE with two cartridges was more likely to obtain favorable products than SPE with one cartridge. In addition, we have compared radiochemical purity, final yield, imaging quality and synthesis time of products purified from SPE with two cartridges to those from HPLC. Radiochemical purity, final yield and imaging quality of SPE with two cartridges were comparable to those from HPLC, while synthesis time of radiotracer from SPE with two cartridges was much shorter than that from HPLC, which indicated that the modified SPE purification was suitable for the clinic use.

In conclusion, the present study demonstrated an improved purification approach for ^18^F-FP-(+)-DTBZ, using SPE with two different cartridges (Sep-Pak PS-2 and Sep-Pak C18). The desired product was prepared in 27 min with a 29 ± 1.8% yield rate. This SPE purification method is highly suited to automatic synthesis for routine clinical applications.

## Data Availability Statement

The raw data supporting the conclusions of this article will be made available by the authors, without undue reservation.

## Ethics Statement

The studies involving human participants were reviewed and approved by the Ethics Committee of The First Hospital of Jilin University. The patients/participants provided their written informed consent to participate in this study. Written informed consent was obtained from the individual(s) for the publication of any potentially identifiable images or data included in this article.

## Author Contributions

YL and HZ designed the study. YD, RS, FG, and YL participated radiosynthesis of tracer. QW and HZ analyzed scans and data. YD and RS wrote and revised the draft. All authors contributed to the article and approved the submitted version.

## Conflict of Interest

The authors declare that the research was conducted in the absence of any commercial or financial relationships that could be construed as a potential conflict of interest.
